# An Ambulatory Method of Identifying Anterior Cruciate Ligament Reconstructed Gait Patterns

**DOI:** 10.3390/s140100887

**Published:** 2014-01-07

**Authors:** Matthew R. Patterson, Eamonn Delahunt, Kevin T. Sweeney, Brian Caulfield

**Affiliations:** 1 Insight Centre for Data Analytics, University College Dublin, Belfield, Dublin 4, Ireland; E-Mails: kevin.sweeney@ucd.ie (K.T.S.); brian.caulfield@ucd.ie (B.C.); 2 School of Public Health, Physiotherapy and Population Science, University College Dublin, Health Sciences Centre, Belfield, Dublin 4, Ireland; E-Mail: eamonn.delahunt@ucd.ie; 3 Institute for Sport and Health, University College Dublin, Dublin 4, Ireland

**Keywords:** locomotion, inertial sensor, gyroscope, knee joint, feature extraction, wearable sensor

## Abstract

The use of inertial sensors to characterize pathological gait has traditionally been based on the calculation of temporal and spatial gait variables from inertial sensor data. This approach has proved successful in the identification of gait deviations in populations where substantial differences from normal gait patterns exist; such as in Parkinsonian gait. However, it is not currently clear if this approach could identify more subtle gait deviations, such as those associated with musculoskeletal injury. This study investigates whether additional analysis of inertial sensor data, based on quantification of gyroscope features of interest, would provide further discriminant capability in this regard. The tested cohort consisted of a group of anterior cruciate ligament reconstructed (ACL-R) females and a group of non-injured female controls, each performed ten walking trials. Gait performance was measured simultaneously using inertial sensors and an optoelectronic marker based system. The ACL-R group displayed kinematic and kinetic deviations from the control group, but no temporal or spatial deviations. This study demonstrates that quantification of gyroscope features can successfully identify changes associated with ACL-R gait, which was not possible using spatial or temporal variables. This finding may also have a role in other clinical applications where small gait deviations exist.

## 1. Introduction

Inertial sensor technology has the potential to bring gait analysis outside of the biomechanics laboratory and allow gait analysis to be more accessible to a wider group of clinicians and researchers on a more regular basis [1,2]. Although current gold standard gait analysis tools, such as stereophotogrammetry and force plates, provide high quality kinematic data, these systems also have many pitfalls such as their cost, their setup time and the fact they are confined to the camera defined collection space. On the contrary, inertial sensors are inexpensive, easy to use when combined with processing software and are not confined to a small collection space [3,4]. Instead of gait analysis being confined to complex biomechanics laboratories, as with traditional gait analysis tools, gait analysis with inertial sensors could take place during regular clinical check-ups, in the home and ubiquitously as people go about their daily lives [5]. Such inertial sensor use could drastically increase the amount of gait data that clinicians and researchers can obtain. The subsequent expansion of available databanks would also allow for enhanced gait rehabilitation programs and more ecologically valid research [6].

Despite the current depth of research in the inertial sensor gait area, the sensors are not used extensively outside of the laboratory environment, save for example as a step counter. This may be partly due to the fact that inertial sensor gait analysis use has been primarily confined to replication of traditional gait analysis metrics; such as temporal, spatial and kinematic data. There is therefore a need for research to look toward using inertial sensor data in innovative ways to provide new clinically meaningful metrics. Despite the availability of the theoretical framework in which inertial sensor data can be integrated to obtain position, it is a well-documented phenomenon that this process results in significant errors [7,8]. It is hypothesized that the use of the raw inertial sensor signal would yield more valid and reliable results as opposed to the error ridden integration to position.

Previous inertial sensor gait research on patient populations has used temporal or spatial outputs from inertial sensors to identify pathological gait patterns, however the pathologies studied have been characterized by large gait deviations [9,10]. Observed differences ranged from 0.5 m in step length for Parkinson patients [10] to 0.6 m in step length for stroke patients [9]. Some pathological conditions, such as anterior crucitate ligament reconstruction or early on-set knee osteoarthritis (OA), may result in gait deviations that do not alter temporal or spatial parameters, but do alter joint angular kinematics and kinetics and can consequently lead to the premature development of degenerative joint disease such as OA. There is a body of research investigating how inertial sensor data can be used to calculate joint angular kinematics, however, the processing techniques are not yet accurate enough to provide clinically useful results [11–13]. Error rates are approximately three degrees when using a robotic knee [11], however these error rates then increase to 7.88 degrees with human use; due to sensor attachment issues and soft tissue movement [13]. In addition, these techniques require the patient to wear multiple sensors. There is a need to consider if clinically useful data can be obtained with fewer sensors, as for ambulatory monitoring applications, decreasing the number of sensors can greatly enhance the usability of the system [14]. The reported errors in estimating joint angular kinematics using inertial sensors are in a range which makes them accurate only for identifying large deviations to the gait pattern. As some abnormal pathological gait patterns have only subtle deviations there is a need for an inertial sensor processing technique that can identify a pathological gait deviation in which no spatio-temporal differences exist, but only subtle kinematic differences exist.

As such, the purpose of this work was to investigate innovative ways by which raw inertial sensor data could be processed to provide clinically useful information. Specifically we want to determine if inertial sensor extracted features can be used to identify abnormal gait patterns in a pathological population with subtle gait pattern differences.

ACL-R gait was chosen as the gait pathology to investigate for three reasons. Firstly, aberrant gait patterns post-ACL-R have been suggested to be a potentially important risk factor for the development of knee OA [15–17]. Secondly, ACL-R gait has not been previously investigated with inertial sensors and thirdly, the deviations between ACL-R and normal, healthy gait patterns are minimal [18–20]. Therefore, ACL-R participants provide a novel test cohort to determine if inertial sensors can be used to detect gait changes that are not obvious to the eye.

## 2. Experimental Section

Seventeen lower limbs of fourteen female athletes constituted the ACL-R group. Of these athletes, three participants had previously ruptured both right and left ACL, thus both lower limbs were included for the analysis in these participants (Table 1). Of the seventeen involved lower limbs analyzed in this work, eight were reconstructed via a hamstring auto-graft surgical procedure, with the remaining being a bone-patellar tendon-bone auto-graft. At the time of testing all athletes were fully engaged in field or court based sports (e.g., Gaelic football, soccer, hockey, basketball) at club or county level and no athlete was undergoing any form of formal rehabilitation. Seventeen female athletes with no previous history of knee joint injury constituted the control group (Table 1). All athletes played field or court based sports (e.g., Gaelic football, soccer, hockey, basketball) at club or county level. Ethical approval for the study was approved by the Universities ethics committee. Before each subject began the study, they were informed of the risks of participation and each read and signed an informed consent form.

Gait data was collected simultaneously using both conventional gait analysis tools as well as inertial sensors. Conventional gait data was obtained using an active marker CODA Motion Analysis System (Charnwood Dynamics, Ltd, Leicestershire, UK) that consisted of three MPX30 cameras sampling at 200 Hz. The system was integrated with two AMTI force plates sampling at 1,000 Hz (Watertown, MA, USA). Motion capture data was obtained using previously described methods [21]. Anthropometric data was obtained for the calculation of internal joint centres at the hip, knee and ankle joints, and included pelvic width (left ASIS to right ASIS), pelvic depth (ASIS to PSIS on right side), knee width and ankle width. Limb lengths of the thigh, shank and foot were also measured with a measuring tape. The markers and marker wands were applied according to manufacturer guidelines by the same investigator on all subjects. Markers were positioned on the lateral aspect of the knee joint line, the lateral malleolus, the heel and the fifth metatarsal head. Wands with anterior and posterior markers were positioned on the pelvis, sacrum, thigh and shank. The markers were fixed to the skin with double sided tape.

Two inertial sensors (Xsens MTx G25, Enchede, The Netherlands), sampling at 100 Hz, were placed on each subject. Each inertial sensor contained a 3-axis accelerometer and gyroscope. For the research presented in this paper, only the sagittal plane shank gyroscope signal was used. The signal range of the gyroscopes were ±1,200 degrees per second. They were placed on the anterior aspect of the tibia, attached to the CODA leg marker, with the centre of the inertial measurement unit at the mid-point between the lateral malleolus and the knee joint centre. The sensors were held in place with double sided tape as well as athletic tape.

After the markers were secured in place, a Chartered Physiotherapist put each subject into a subtalar neutral standing position prior to collection of a neutral stance trial. This was performed to align the participant with the laboratory coordinate system and to function as a reference position for subsequent kinematic and kinetic analysis. Prior to performing the trial, each subject completed several practice walking trials through the laboratory walkway. This allowed the subjects to get accustomed to the markers as well as allowing a starting point to be identified so that the subjects would contact the force plates in normal stride. Subjects walked barefoot across the 15 m walkway at their self-selected *normal* walking speed. To ensure normal walking throughout each trial, the subjects were not made aware of the presence of the force plates until the data collection was completed. Any trials in which there were not two consecutive foot strikes onto the force plates were discarded. Ten *clean* gait cycles, in which two full foot strikes onto the force plates were detected, were saved. The employed measurement system has been previously shown to be reliable when using the same marker set up and ten gait trials [21].

### 2.1. Data Processing of Inertial Sensors

Heel strike (HS) and toe-off (TO) points were found in the inertial sensor data based on a previously validated algorithm [22]. From these gait events, four temporal variables were calculated for each trial; gait cycle duration, stance time, swing time and double support time.

**Figure 1.**
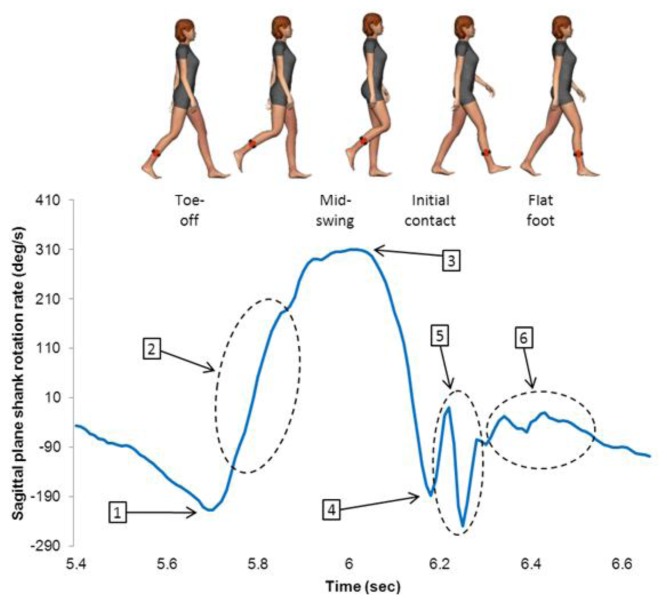
The sagittal plane shank gyroscope signal over a single gait cycle. The quantified features from each gait cycle are numbered on the graph. 1—minimum value at TO, 2—rate of change during initial swing, 3—peak shank rotation rate during swing, 4—minimum value at IC, 5—post-HS shank variance and 6—mid-stance variance.

A data-driven approach was used to determine if data from the sagittal plane shank gyroscope could be used to differentiate between ACL-R and healthy control gait patterns [23]. Six gyroscope features during each gait cycle on each foot were quantified from the sagittal plane gyroscope (Figure 1). These were selected based on previous research which indicated that the sagittal plane shank rotation rate signal can be used to identify pathological gait patterns [10]. Minimum value at TO represents the large minimum prior to peak rotation rate at mid-swing. Rate of change during initial swing was determined by calculating the rate of change of the sagittal plane shank rotation rate from TO until 25% of the swing phase. Peak shank rotation rate during swing is the maximum rotation rate the shank achieves during the swing phase. Peak negative rotation rate at HS occurs at the first minimum following the maximum rotation rate during mid-swing. Post-HS shank variance was calculated by taking the variance of the gyroscope signal from HS until 35% of the stance phase. Mid-stance variance was calculated by taking the variance of the gyroscope signal from 35% stance to 75% stance.

Inertial sensor outputs (temporal and extracted features) from each steady state gait cycle were averaged for each trial. Values from each trial were averaged to obtain one value for each variable for every subject. Values from each subject were averaged to obtain one value for the ACL-R group and one value for the control group. For ACL-R participants, data from the injured legs were used. For control subjects, either the right or left leg was used to allow for an equal number of right and left legs in both the ACL-R and control groups. Independent samples t-tests were used to test for differences between groups in the temporal data as well as the quantified gyroscope variables.

### 2.2. Data Processing of Conventional Gait Data

Gait speed was calculated by dividing the stride length by the stride duration of the right heel marker, which was obtained from the motion capture software. Knee angular kinematic and kinetic data from each trial was accumulated over the entire stride. The first initial contact was found in the force plate data as a 10 N crossing of the vertical ground reaction force. The next initial contact was found in the kinematic data using a previously validated method [24]. Each trial of each participant was normalized to 400 samples and averaged over all ten trials. The averaged curves from each subject were averaged over each group to obtain the comparison curves between the ACL-R and the control groups. Independent two-sided t-tests were used to test for significant differences between magnitudes of the group means time averaged profiles for each variable recorded during the stride. This technique has been used previously [25,26]. The level of significance was set at *p* < 0.05.

Peak knee adduction moment during early stance and normalized peak knee adduction moment (%Bw-ht) [27] during early stance were compared using an independent samples one sided t-test (PASW Statistics, 24 Version 18.0, IBM Corporation, Chicago, IL, USA). The level of significance was set at *p* < 0.05. Associated effect sizes (eta squared) were calculated using the formula described in Pallant [28]: t^2^/[t^2^ + (N1 + N2 – 2)] and quantified according to Cohen [29] as 0.01 = small effect size, 0.06 = medium effect size and 0.14 = large effect size. Pearson product correlations and subsequent significance values were used to investigate the relationship between the six features which were extracted from the gyroscope signal.

## 3. Results and Discussion

### 3.1. Temporal Features and Gyroscope Extracted Features

As can be observed from Table 2, there were no significant differences in temporal parameters between the ACL-R group and the control group. However, a significant difference was found between the groups in three of the gyroscope variables: peak rotation rate at mid-swing for the ACL-R group was significantly lower than the control group; minimum rotation rate at IC for the ACL-R group was significantly lower than the control group; post-HS shank adjustments for the ACL-R group were significantly lower than the control group.

Table 3 presents eta squared, cohen's D and 95% confidence intervals of the difference for all gyroscope extracted features with significance values less than 0.05 when comparing between the ACL-R group and the control group.

Table 4 presents Pearson product correlation information between each of the six gyroscope extracted features.

### 3.2. Conventional Gait Features

**Figure 2.**
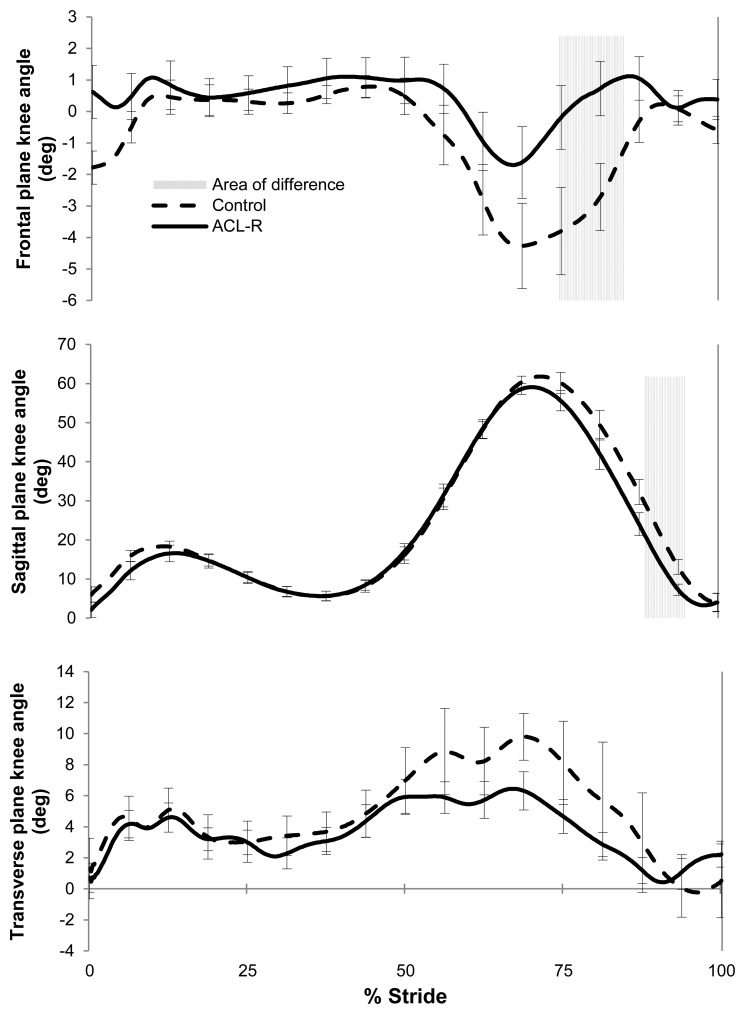
3D knee angular kinematic time averaged profiles for the ACL-R group and the control group normalized over the stride. Segments with significant differences between the ACL-R and control groups are shown in the shaded sections.

Peak knee adduction moment was lower for the ACL-R group (N = 17, M = 0.31 Nm/kg·m, SD = 0.08) than for the control group (N = 17, M = 0.41 Nm/kg·m, SD = 0.12; t(32) = 2.483, p = 0.010, one tailed, Figure 2), with an associated large effect size (eta squared = 0.16). Normalized knee adduction moment was also lower for the ACL-R group (N = 17, M = 2.86, SD = 1.01) than for the control group (N = 17, M = 3.89, SD = 1.13; t(32) = 2.770, p = 0.005, one tailed), with an observed large effect size (eta squared = 0.19).

A comparison of time averaged profiles for knee angular displacement in coronal, frontal and sagittal planes showed that there were significant differences in knee joint kinematics between the groups during the swing phase (Figure 2). No differences were observed between groups during the stance phase of the gait cycle (Figure 2). The ACL-R participants displayed a more extended and more adducted knee position during swing phase than the control subjects.

## 4. Conclusions/Outlook

This work extends the current use of inertial sensors to analyze gait patterns by extracting features from a gyroscope on the shank which are capable of identifying the presence of a pathological gait condition that is not obvious to the naked eye. Inertial sensors have been used previously to identify gait pathologies in which there are large temporal or spatial abnormalities in gait patterns [9,10,30]. However, since there were no significant temporal or spatial deviations in this ACL-R group, the authors quantified features from a shank mounted gyroscope to differentiate between ACL-R and healthy control gait patterns. Due to the potential association between aberrant ACL-R gait patterns and early onset of knee joint OA [15–17], the results of the present study suggest that inertial sensor extracted features may one day be applicable in the clinical domain as a screening tool to easily identify patients who have potentially harmful gait patterns; although more work is required to prove this hypothesis. Inertial sensors are a valuable tool for gait analysis due to their low cost, portability and ease of use compared to conventional gait analysis tools. Regular inertial sensor use for gait analysis would result in a significant increase in the amount of gait data that clinicians and researchers can collect.

The conventional gait analysis tools used in this study indicate that there were subtle gait pattern deviations between the ACL-R and control groups however there were no temporal or spatial differences in the gait patterns between groups (Tables 1 and 2). There were small knee joint angular kinematic differences between the groups; for example ACL-R participants displayed a more extended and adducted knee position during swing (Figure 2). Such a position may apply an increased translational shear stress on the knee joint. The ACL-R participants also displayed a significantly lower peak knee adduction moment. Current state of the art inertial sensor processing techniques can accurately determine spatial and temporal gait metrics, but not joint angular kinematics. Thus, this paper proposed a novel method of processing inertial sensor data to detect ACL-R gait patterns; which involved extracting features from the sagittal plane shank gyroscope signal.

The findings of this work and previous work [10] on inertial sensor extracted features suggest that there may be a relationship between peak shank rotation rate during swing and harmful gait patterns; further work is required. Previous published work has reported statistically significant differences of 0.2 and 0.4 s in gait cycle time between two different Parkinson patient groups compared to healthy controls [10]. In the present study a non-significant 0.02 s difference was found in gait cycle time between the ACL-R group and the control group (Table 5). However, a significant difference did exist in the peak shank rotation rate during swing. The ACL-R patients in the present study had values that were significantly less than an age and activity matched control group. These values were similar to those values reported in an elderly control group in a previous study [10]. Having a decreased peak shank rotation rate during swing alone is not likely to be a problem, however it is an interesting finding that two very different pathological populations who have been shown to be at an elevated risk of knee joint OA development displayed similar peak shank rotation rate values during swing. ACL-R patients are at a higher risk of developing knee joint OA and a potential mechanism of this is thought to be abnormal locomotion patterns [15–17]. Elderly patients are also at a high risk of developing knee OA from abnormal locomotion patterns [31]. Perhaps, a reduced peak shank rotation rate during swing is a characteristic of gait in subjects who have gait patterns that may potentially cause long-term knee joint degeneration. The results of this study do not confirm this, but these results combined with previous research suggest that this inertial sensor extracted feature may be an important marker of harmful gait patterns. More work is warranted to thoroughly investigate this potential relationship.

All three of the extracted features which were significantly different between the ACL-R and the control groups were of lower magnitude in the ACL-R group (Table 2). Based on similar relationships between knee kinematics and shank rotation rate in previous research [10,32], this decrease in magnitude of shank rotation rate at IC (when the knee is extended) may be related to some motor strategy to limit strain on the ACL. This adaptation strategy may have also resulted in the significantly lower shank rotation rate variance seen in initial stance of the ACL-R group compared to the control group. As the peak value at IC was decreased, the rotation rate had less of a peak value to recover from during initial stance, resulting in the lower shank rotation rate variance seen during initial stance of the ACL-R group. Post-IC shank rotation rate variance is most likely linked to the body beginning to move over the foot at the start of stance.

Table 4 implies that there is a close relationship (0.79, −0.83 and −0.91 Pearson product correlation) between all of the gyroscope extracted features that are significantly different between the ACL-R group and the control group. This suggests that they are providing similar information regarding the differences observed in ACL-R gait patterns. Perhaps, if future biomechanical research conclusively proves that knee adduction moment is a strong predictor of knee joint degeneration over time, extracted features from a single inertial sensor could be used to assess risk of joint degeneration. Such an approach could utilize multiple regression techniques in which these correlations are useful to determine variables providing redundant information which can therefore be dropped from the multiple regression equation. The advantage of estimating joint moments in the future using one inertial sensor is that joint moment calculations require the measurement of angular kinematics as well as ground reaction forces, which require a significant amount of measurement equipment. Using one inertial sensor means it would be much easier to obtain the complex moment data, thus allowing the collection of an important biomechanical marker outside of the constraining laboratory environment.

Based on the fact that epidemiological research has shown that ACL-R patients are five times more likely to develop knee joint OA than the general population [33] and these extracted features are different in an ACL-R population, these extracted features could be useful at identifying patients who have abnormal gait patterns that may result in long-term knee joint degenerative disease. This hypothesis suggests that inertial sensors and their extracted features might one day be useful in clinical practice for use as a screening tool for pathological populations in which only subtle gait deviations exist. In such a scenario, an inertial sensor could be mounted on the shank and if the feature of interest is in an abnormal range, it would indicate to the clinician that that patient should undergo a more in-depth gait analysis using the more costly and time consuming traditional gait tools. Such a screening tool would allow a larger number of patients to be analyzed at a lower cost to both the patient and the health-care system.

An *a priori* sample size calculation was not possible, as previous work had not considered inertial sensor extracted features in an ACL-R population. Post-hoc analysis showed that the differences between groups for the peak shank rotation rate during swing and the shank rotation rate at IC both seem to be real observed effects based on the fact that their effect sizes are large, Cohen's D are over 0.80 and their observed powers are both over 0.90 (Table 3). The difference between groups for post-IC shank variance does not have a high level of observed power and only a moderate effect size, providing less confidence that the difference seen between groups for that variable was a real observed effect (Table 3).

A limitation of this study is that it does not conclusively prove any link between inertial sensor extracted features and the presence of abnormal movement patterns which may lead to knee joint degeneration over time. Estimating knee joint OA development is not a simple issue, but a complex, multifaceted problem that is well beyond the scope of one cross-sectional study.

The main contribution of this work is that a novel processing method has been employed to obtain clinically useful gait information from a musculoskeletal pathology which results in only subtle gait pattern deviations.

## Figures and Tables

**Table 1.** Anthropometric, gait velocity and surgical data. Averages are presented with standard deviations in brackets. Differences between group means are non-significant (*p* > 0.05) except for age (*p* < 0.01).

	**Age (y)**	**Height (m)**	**Weight (kg)**	**Walking Speed (m/s)**	**Time Since Surgery** **(year)**
Control	20.8 (1.17)	1.65 (0.06)	64.7 (7.06)	1.42 (0.13)	
ACL-R	23.7 (3.12)	1.64 (0.05)	64.9 (9.02)	1.37 (0.13)	3.50 (3.25)

**Table 2.** Temporal gait parameters compared between the ACL-R and control groups. Average values are presented with standard deviations in brackets.

**Temporal Variable**	**ACL-R**	**Control**	**t**	**Sig**
Gait cycle (s)	1.008 (0.063)	0.975 (0.036)	1.833	0.076
Stance time (s)	0.570 (0.047)	0.544 (0.026)	1.973	0.057
Swing time (s)	0.438 (0.037)	0.434 (0.025)	0.382	0.765
Double support time (s)	0.060 (0.025)	0.058 (0.015)	0.334	0.741

Gyroscope extracted features				

Shank rotation rate at TO (rad/s)	−4.293 (0.753)	−4.481 (0.674)	0.768	0.448
Shank rate of change during initial swing (rad/s/s)	0.767 (0.212)	0.762 (0.224)	0.063	0.950
Peak shank rotation rate during swing (rad/s)	6.935 (0.695)	7.517 (0.562)	−2.680	0.012 *
Shank rotation rate at IC (rad/s)	−3.452 (0.614)	−4.105 (0.699)	2.893	0.007 *
Post-IC shank rotation rate variance (rad/s)	0.898 (0.534)	1.246 (0.434)	−2.082	0.045 *
Shank rotation rate variance during mid-stance (rad/s)	0.237 (0.104)	0.321 (0.200)	−1.551	0.131

* significant difference between the groups (*p* < 0.05).

**Table 3.** Eta squared, cohen's D and 95% confidence intervals of the difference for all gyroscope extracted features with significance values less than 0.05.

	**Eta** **Squared**	**Cohen's** **D**	**Power**	**95% Confidence Intervals of** **the Difference**	

				**Lower**	**Upper**
Peak shank rotation rate during swing	0.184 (large)	0.921	0.740	−1.023	−0.140
Peak shank rotation rate at IC	0.207 (large)	0.993	0.801	0.193	1.113
Post-IC shank rotation rate variance	0.120 (moderate)	0.715	0.525	−0.688	−0.008

**Table 4.** Pearson product correlations and significance values (presented in brackets) between each of the gyroscope extracted features.

	**Shank Rotation Rate at TO**	**Shank Rate of Change During Initial Swing**	**Peak Shank Rotation Rate During Swing**	**Shank Rotation Rate at IC**	**Post-IC Shank Rotation Rate Variance**	**Shank Rotation Rate Variance During Mid-Stance**
Shank rotation rate at TO	-	−0.83 (< 0.01)	−0.56 (< 0.01)	0.32 (< 0.063)	−0.47 (< 0.01)	−0.53 (< 0.01)
Shank rate of change during initial swing	-	-	0.44 (< 0.01)	−0.30 (0.086)	−0.40 (< 0.018)	0.51 (< 0.01)
Peak shank rotation rate during swing	-	-	-	−0.83 (< 0.01)	0.79 (< 0.01)	0.42 (< 0.01)
Shank rotation rate at IC	-	-	-	-	−0.91 (< 0.01)	−0.44 (< 0.01)
Post-IC shank rotation rate variance	-	-	-	-	-	0.42 (0.012)

**Table 5.** Peak shank rotation rate during mid-swing comparison. Mean values are presented with standard deviations in brackets.

**Study**	**Group**	**Average** **Age (year)**	**Gait Cycle** **Time (s)**	**Peak Shank Rotation Rate** **During Mid-Swing (deg/s)**
Salarian *et al.* [10]	Parkinson stim on	61.5	1.4 (0.6)	225.2 (103.5)
	Parkinson stim off	61.5	1.2 (0.2)	275.4 (110.0)
	Control	63.6	1.0 (0.1)	386.3 (40.1)

Current study	ACL-R	20.8	1.0 (0.063)	397.6 (40.1)
	Control	22.6	0.98 (0.034)	430.9 (32.1)
